# Autonomic Dysfunction in Autism Spectrum Disorder

**DOI:** 10.3389/fnint.2021.787037

**Published:** 2021-12-30

**Authors:** Andrew P. Owens, Christopher J. Mathias, Valeria Iodice

**Affiliations:** ^1^Department of Old Age Psychiatry, King’s College London, Institute of Psychiatry, Psychology and Neuroscience, London, United Kingdom; ^2^Autonomic Unit, National Hospital Neurology and Neurosurgery, UCLH NHS Trust, London, United Kingdom; ^3^UCL Queen Square Institute of Neurology, University College London, London, United Kingdom; ^4^Neurovascular Medicine Unit, Lindo Wing, St Mary’s Hospital, Imperial College NHS Healthcare Trust, London, United Kingdom

**Keywords:** autism, autonomic nervous system, postural tachycardia syndrome, syncope, hyperhidriosis, hypermobile ehlers danlos syndrome, dysautonomia

## Abstract

**Background:** There have been previous reports of enhanced sympathoexcitation in autism spectrum disorder (ASD). However, there has been no formal investigation of autonomic dysfunction in ASD. Also, the joint hypermobile form of Ehlers-Danlos syndrome (hE-DS) that maybe overrepresented in ASD and orthostatic related autonomic dysfunction. This study examined the comorbidity of ASD, autonomic dysfunction and hE-DS in two UK autonomic national referral centers. Proven, documented and globally accepted clinical autonomic investigations were used to assess neuro-cardiovascular autonomic function in a cohort of ASD subjects and in age-matched healthy controls.

**Methods:** Clinical data from 28 referrals with a confirmed diagnosis of ASD over a 10-year period were compared with 19 age-matched healthy controls. Autonomic function was determined using methods established in the centers previously described in detail.

**Results:** 20/28 ASD had a diagnosed autonomic condition; 9 had the postural tachycardia syndrome (PoTS), 4 PoTS and vasovagal syncope (VVS), 3 experienced presyncope, 1 essential hyperhidrosis, 1 orthostatic hypotension, 1 VVS alone and 1 a combination of PoTS, VVS and essential hyperhidrosis. 16/20 ASD with autonomic dysfunction had hE-DS. In ASD, basal heart rate and responses to orthostatic tests of autonomic function were elevated, supporting previous findings of increased sympathoexcitation. However, sympathetic vasoconstriction was impaired in ASD.

**Conclusion:** Intermittent neuro-cardiovascular autonomic dysfunction affecting heart rate and blood pressure was over-represented in ASD. There is a strong association with hE-DS. Autonomic dysfunction may further impair quality of life in ASD, particularly in those unable to adequately express their experience of autonomic symptoms.

## Introduction

Homeostatic regulation is facilitated by the autonomic nervous system (ANS) and its ability to mediate activity of bodily organs especially the heart and blood vessels via peripheral efferent nerves. The term “autonomic” derives from the fact that its function is largely beyond conscious control. The ANS has sympathetic nervous system (SNS) and the parasympathetic nervous system (PNS) efferent pathways. The SNS increases effector organ activity predominantly via the catecholamines, such as noradrenaline (NA) at the neuro-effector junction. The PNS promotes downregulation of effector organ activity, through neuro-transmitters such as acetylcholine, slowing heart rate and facilitating food digestion by its effects on the gastrointestinal tract.

The literature investigating autonomic function in autism spectrum disorder (ASD), particularly during behavioral studies, report a prevalence of enhanced sympathoexcitation (increased cardiovascular and sudomotor activity) (Althaus et al., 2004; Bal et al., 2010; Porges et al., 2013). Autonomic abnormalities have also been reported in relation to psychological factors using self-report measures (Ming et al., 2011) by those with ASD. However, studies into pupillary responses and cardiovagal function have produced contradictory findings. Some studies have found smaller pupil size in ASD (Martineau et al., 2011), unlike others with increased pupil size (Anderson et al., 2013). In comparison to controls and on cardiovagal function, some studies have found vagally-mediated cardiac modulation to be increased in ASD (Toichi and Kamio, 2003), whilst others have found the opposite (Bal et al., 2010). Autonomic dysregulation has been proposed by some as a contributing factor to the neuropsychiatric symptoms in ASD (Hutt et al., 1964; Porges et al., 1996, 2013). However, no study has yet investigated autonomic function or dysfunction (dysautonomia) in ASD. The purpose of this study was to use clinical laboratory investigations to determine if there was autonomic dysfunction or an autonomic disorder in a cohort with ASD, in comparison with age-matched healthy controls.

Autonomic dysfunction can present as a primary or secondary feature, complicating neurological, cardiovascular, endocrine, metabolic and genetic disorders. There are:

• “fixed” forms, with autonomic damage and failure that usually is irreversible (such as spinal cord injury), and potentially progressive (as in Parkinson’s disease with autonomic failure and multiple system atrophy) (Bannister and Mathias, 2013), the latter usually occurring in middle-to-late adulthood;
• “intermittent” forms, with no obvious damage to autonomic nerves, but a transient dysregulation of otherwise normal autonomic function due to a specific allostatic stimulus, such as standing upright as in the postural tachycardia syndrome (PoTS) or with temperature change (thermoregulatory dysfunction). Intermittent autonomic disorders can present during adolescence and early adulthood, often with a positive family history (Mathias et al., 1998, 2012; Kaufmann et al., 2003).

Fixed forms of autonomic disorders with autonomic failure are characteristically due to trauma to the ANS or neurodegeneration (spinal cord injury or Parkinsonism), and thus intermittent autonomic dysfunction is more likely to occur in ASD. A common consequence of orthostatic intolerance is fainting (syncope). Autonomic (neurally) mediated syncope (AMS) usually occurs due to a transient breakdown of homeostatic autonomic reflex arcs during standing and may be due to:

• Situational syncope—attributable to a specific stimulus, such as straining during weightlifting or playing a wind instrument, often increasing intrathoracic pressure;
• Vasovagal syncope (VVS)—the most common (80% of cases) form of syncope. In VVS, sympathoexcitation (increased heart rate and sweating) can precede the drop in blood pressure due to vasodilatation (due to sympathetic withdrawal) and bradycardia (due to increased vagal/parasympathoexcitation);
• Carotid sinus sensitivity—typically in those aged > 50 years from aberrant baroreceptor overactivation in nerves around the carotid artery.

The most common form of orthostatic intolerance probably is PoTS, defined by a heart rate increase of > 30 BPM or heart rate of > 120 BPM with symptoms of orthostatic intolerance (dizziness, palpitations), but without orthostatic hypotension (Owens et al., 2017) when standing upright or within 10 min of orthostasis or head-up tilt (HUT) (Freeman et al., 2011). Some consider PoTS under hyperadrenergic or neuropathic phenotypes (Benarroch, 2012), although this often is not readily determined on clinical evaluation or testing. Infection, deconditioning, hypovolemia and impaired cerebral autoregulation have been implicated as triggers in PoTS or its pathophysiology.

Essential hyperhidrosis is the most common form of intermittent autonomic thermodysregulation and is defined by profuse and uncontrollable sweating exceeding that needed to maintain thermostasis, often causing significant functional impairment, and without a definitive cause. Essential hyperhidrosis can present as excessively local, on the palms, soles and axillae, or can be generalized. The prevalence of essential hyperhidrosis has been estimated as 2–3% (Ocon et al., 2009; Lai et al., 2015)but the etiology remains uncertain. Once a secondary cause of hyperhidrosis has been eliminated (e.g., drug side-effect), sudomotor activity can be measured by a thermoregulatory sweat test (Moraites et al., 2014).

Orthostatic intolerance is often associated with the joint hypermobility syndrome and Ehlers-Danlos syndrome (E-DS) Type III, now considered as the joint hypermobile form of E-DS (hE-DS). It is a heritable rheumatological condition characterized with variant collagen distribution across connective tissue, but without an identified gene as yet. hE-DS appears overrepresented in ASD (Eccles et al., 2014) and may predispose to orthostatic intolerance and PoTS (Mathias et al., 2012; Eccles et al., 2015). Despite the co-occurrence of hE-DS in ASD and forms of orthostatic intolerance, a detailed investigation into the presence of autonomic disorders in ASD has not yet been undertaken. Autonomic dysfunction in those with ASD may further impair morbidity and quality of life (QoL), and this study examined the potential comorbidity of ASD and clinical autonomic dysfunction, focusing also on intermittent autonomic disorders, in two UK national referral centers for autonomic disorders that have worked in unison (the Autonomic Unit at the National Hospital for Neurology and Neurosurgery, University College London Hospitals, Queen Square, and the Pickering Unit at St Mary’s Hospital, Imperial College Healthcare NHS Trust). There also was an emphasis on clinical features that may further aid our understanding of autonomic function studied in detail in ASD, which previously has not been addressed.

## Materials and Methods

### Ethics

National (London—Harrow Research Ethics Committee) and institutional (University College London and Imperial College London) ethics were acquired for the examination of the medical records of all patients referred over a 10-year period (2007–2017) with a confirmed diagnosis of ASD.

### Participants

We identified 28 autonomic evaluation referrals with a confirmed diagnosis of ASD (aged 29 ± 8.66, 12 female, 18 male). ASD data was compared to existing age-matched healthy control data (*n* = 19, mean age 29 ± 12.85, 10 female, 9 male). None of the healthy control group had a history of cranial injury, neurological disorder, cognitive impairment or learning disability. The controls were recruited from Imperial College London, University College London and members of the local community. All participants provided written informed consent.

### Medication

Medication of relevance to autonomic conditions in those with ASD is detailed in Table 1 of the results section.

**TABLE 1:** Overview of ASD referrals with a diagnosable form of autonomic dysfunction.

Autonomic diagnosis	Hypermobile form of Ehlers-danlos syndrome (hE-DS)	Medication
9 PoTS (3 female, 6 male)	7/9 (78%)	8 analgesic
4 Comorbid PoTS and VVS (4 female)	4/4 (100%)	7 antidepressant
3 Presyncope (2 female, 1 male)	3/3 (100%)	5 anti-epileptic drug
1 VVS (male)	1/1 (100%)	4 proton pump inhibitor 4 anti-inflammatory 4 antipsychotic
1 Generalized EH (female)	–	3 Salbutamol inhaler 3 migraine treatment 3 anti-nausea
1 orthostatic hypotension (female)	1/1 (100%)	2 anti-bladder spasmodic 2 constipation medication 2 antihistaminics
1 Comorbid PoTS, VVS, and craniofacial EH (male)	–	Medication
20/25 (80%)	16/20 (80%)	

*EH, essential hyperhidrosis; PoTS, postural tachycardia syndrome; VVS, vasovagal syncope. The numbers and percentage with an autonomic diagnosis and hE-DS are indicated below.*

### Autonomic Assessment

Heart rate and the electrocardiogram (ECG) were continually monitored online (PowerLab 16/30/ECG (Bioamp) (AD Instruments, Oxford, United Kingdom) and analyzed offline (Labchart 7). Blood pressure was continually recorded using digital photoplethysmography (Finometer, Smart Medical, Gloucestershire, United Kingdom) and heart rate and blood pressure measures were taken using automated sphygmomanometry (Dinamap Pro400V2, GE Healthcare, Buckinghamshire, United Kingdom). The evaluations were performed during the tests outlined, and performed in the laboratories (with identical equipment) in the two autonomic laboratories that were an integral part of the national referral centers.

Before autonomic testing, each patient underwent detailed clinical evaluation (history/examination) by a consultant autonomic physician who documented relevant information, to include family history of orthostatic intolerance. This included relevant autonomic symptoms (such as postural dizziness and palpitations) and factors worsening intolerance (such as standing still, food, exertion, heat and in female the menstrua; period). Included was assessment for hE-DS, based on clinical examination and current criteria (Castori et al., 2017). Historical or current psychiatric conditions diagnosed by a mental healthcare professional were recorded. Informed consent was received from each patient prior to testing and, if tolerable, patients were asked to discontinue any medications that may affect autonomic function 24–48 h prior to testing and abstain from caffeine, nicotine and vigorous exercise 24 h before testing. Established and validated clinical protocols (Bannister and Mathias, 2013; Mathias et al., 2013) assessed:

• Sympathetic vasoconstriction during mental arithmetic (1 min), isometric exercise (3 min) and cutaneous cold pressor responses (90 s). Pressor maneuvers, including isometric (hand-grip) exercise, cutaneous cold application, deep breathing and mental arithmetic provide an index of sympathetic nerve activity (SNA) and induce autonomic cardiovascular changes, particularly in blood pressure, which is regulated via the SNS. Isometric and cutaneous cold pressor stimuli raise blood pressure via activation of sympathetic efferent nerve pathways and provide more responsive data in comparison to mental arithmetic or other pressor tests. Peripheral receptors are activated but in both cutaneous cold or isometric exercise tests there is an important central command (isometric) or nociceptive (cold) role, which is more pronounced in isometric exercise leading to a greater increase in SNA compared to the cold pressor test;
• Cardiac vagal function during respiratory sinus arrhythmia (RSA, 1 min). In normal subjects there is an increase in heat rate during inspiration and a decrease in HR during expiration, known as “respiratory sinus arrhythmia” (RSA), which is a measure of the functional endpoint of cardioinhibitory vagal fibers emanating from the nucleus ambiguus in the brainstem. In this study cardiovagal function was assessed by subtracting the minimum heart rate response from the maximum heart rate response to deep breathing averaged over a minimum of 5 breathing cycles (HRDB) (Bannister and Mathias, 2013; Mathias et al., 2013);
• Baroreflex cardiovagal and adrenergic function using the Valsalva maneuver, measures central and peripheral baroreflex pathways. The Valsalva maneuver involves forced expiratory pressure (40 mmHg) against a blocked airway for 10–15 s (repeated 2/3 times). The increase in intrathoracic pressure causes heart rate and blood pressure changes that can be used to calculate indices of sympathetic and parasympathetic activity;
• Orthostatic tolerance, baroreflex and adrenergic function during head-up tilt (HUT, 9 min). HUT is used to diagnose various forms of orthostatic intolerance. In healthy subjects the initial blood pressure fall induced by HUT should recover within 60 s as decreased venous return to the heart causes reduced stroke volume and cardiac output, activating arterial baroreceptors and cardiopulmonary mechanoreceptors that signal autonomic brain centers to increase sympathetic nerve activity, raising HR and causing vasoconstriction in various vascular beds to compensate for gravitational demands. In normal subjects in whom the baroreflex is intact, HUT of 45–90° should not provoke a prolonged fall in BP. HUT is a valuable investigation that can be terminated if syncopal, pre-syncopal or other OI-related symptoms are recorded or reported by the participant.

During orthostatic challenge, lower extremities were examined for signs of vascular pooling, such as mottling and cyanotic discoloration, indicative of impaired venous return.

### Statistical Analysis

Statistical analysis was performed using SPSS (version 25). Descriptive statistics are presented as mean (± 1 SD) for normally distributed data. Data were tested (Shapiro-Wilk test) for normality and homogeneity of variance. Quantitative variables were compared at single time points by independent *t*-tests for two groups to compare the results of clinical autonomic testing in patients with ASD and age-matched healthy controls. When appropriate, non-parametric tests were used to compare groups (Mann-Whitney *U* Test). Statistical significance was specified as a 2-tailed *p*-value of < 0.05.

## Results

### Clinical Data

Of the 28 ASD group, 3 were unable to complete the full test protocol due to anxiety, joint pain or apneic episodes but their data was analyzed until the cessation of testing. Group mean values for each autonomic test are shown in Table 2.

**TABLE 2:** Summary of autonomic test data in controls and ASD.

*Groups*	Autonomic domain	Baseline	Isometric exercise	Cold pressor	Mental arithmetic	Head up tilt
*Healthy controls*	Heart rate (BPM)	63 ± 8.49	77 ± 10.24	61 ± 8.97	62 ± 8.11	78 ± 8.99
	SBP (mmHg)	122 ± 12.49	146 ± 13.26	133 ± 17.35	118 ± 10.62	116 ± 16.51
	DBP (mmHg)	66 ± 5.84	88 ± 9.93	61 ± 8.97	65 ± 9.08	69 ± 10.31
*Autism spectrum disorder*	Heart rate (BPM)	77 ± 14.66*	87 ± 17.27*	79 ± 16.52*	81 ± 13.07*	99 ± 16.46*
	SBP (mmHg)	118 ± 11.02	132 ± 45*	126 ± 10.72*	118 ± 10.55*	118 ± 12.5*
	DBP (mmHg)	69 ± 8.37	78 ± 7.17*	75 ± 7.12	70 ± 9.48	74 ± 8.11

(± 1 SD), Descriptive statistics are presented as mean; HR, heart rate (bpm); SBP, systolic blood pressure (mmHg), DBP, diastolic blood pressure (mmHg). Significant difference in ASD from controls indicated by*. The bold values refer to significant findings.

### Baseline

**FIGURE 1:**
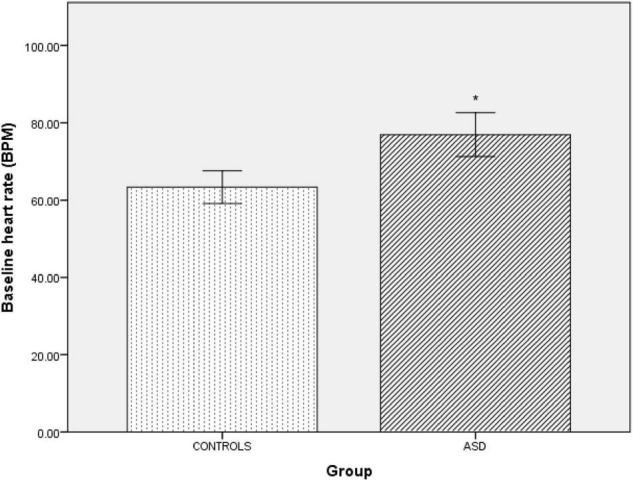
*|* Baseline supine heart rate in controls and ASD. BPM, beats per minute. *Refer to a statistically significant finding.

The ASD group had a significantly [*t*(42) = –4.69, *p* = 0.001] higher supine resting heart rate (77 bpm ± 14.66) than the control group (63 bpm ± 8.49, Figure 1). Between-group blood pressure measures were comparable.

### Isometric Exercise

**FIGURE 2:**
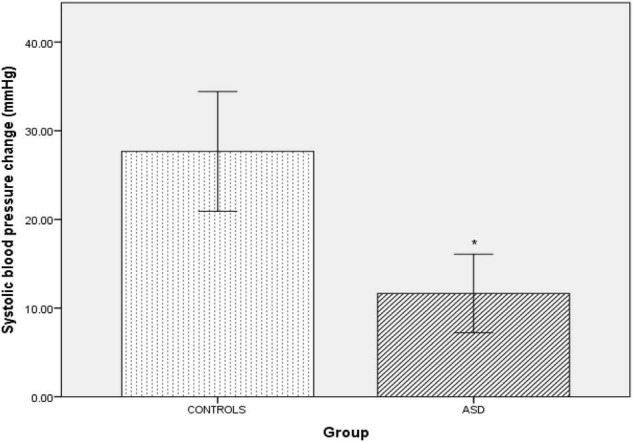
Changes in mean systolic blood pressure from baseline during isometric exercise in controls and ASD. mmHg, millimeters of mercury. *Refer to a statistically significant finding.

Pressor stimuli, such as isometric exercise, cutaneous cold application and mental arithmetic, examine SNS vasoconstriction. In the ASD group, there was a significantly [*t*(36) = 4.25, *p* =<0.001) blunted (Δ11 mmHg ± 9.44) systolic blood pressure response to isometric exercise compared to the control group (Δ15 mmHg ± 13.57, Figure 2).

**FIGURE 3:**
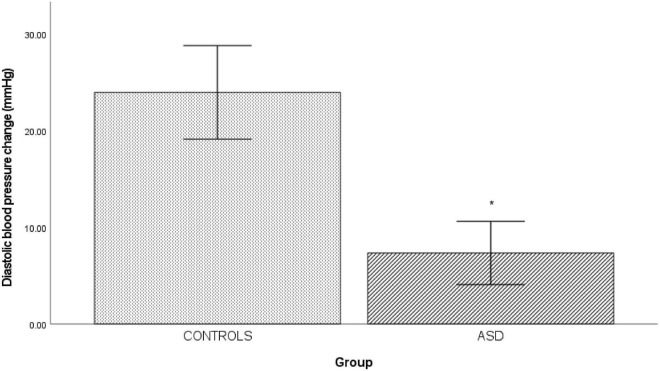
Changes in mean diastolic blood pressure from baseline during isometric exercise in controls and ASD. mmHg, millimeters of mercury. *Refer to a statistically significant finding.

In the ASD group, there was a significantly [*t*(37) = 6.12, *p* = 0.001] blunted diastolic blood pressure response (Δ 7.33 mmHg ± 7.19) to isometric exercise compared to the control group (Δ 23.44 mmHg ± 9.73, Figure 3).

### Cold Pressor Test

**FIGURE 4:**
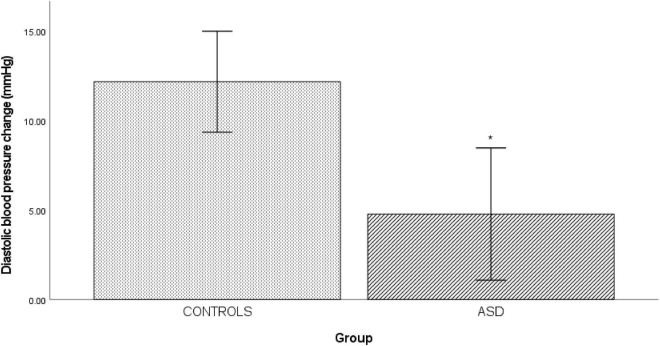
Changes in mean diastolic blood pressure from baseline during cold pressor in controls and ASD. mmHg, millimeters of mercury. *Refer to a statistically significant finding.

In the ASD group, there was a significantly [*t*(38) = 3.20, *p* = 0.003] blunted diastolic blood pressure response (Δ 4.77 mmHg ± 8.34) to isometric exercise compared to the control group (Δ 12.17 mmHg ± 5.67, Figure 4).

### Mental Arithmetic

**FIGURE 5:**
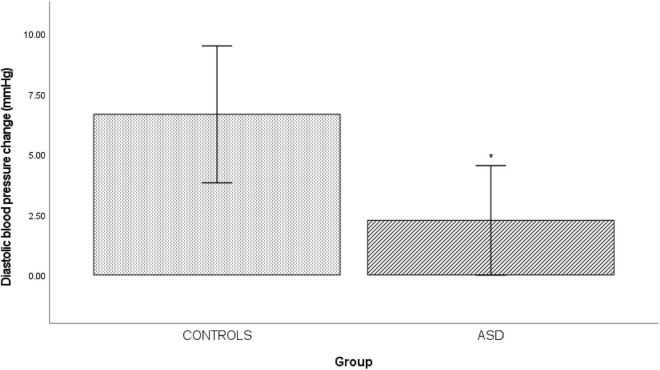
Changes in mean diastolic blood pressure from baseline during mental arithmetic in controls and ASD. mmHg, millimete*r*s of mercury. *Refer to a statistically significant finding.

In the ASD group there was a significantly [*t*(33) = 0.333, *p* = 0.014] blunted diastolic blood pressure response (Δ 2.26 mmHg ± 5.23) to mental arithmetic compared to the control group (Figure 5).

### Head-Up Tilt

**FIGURE 6:**
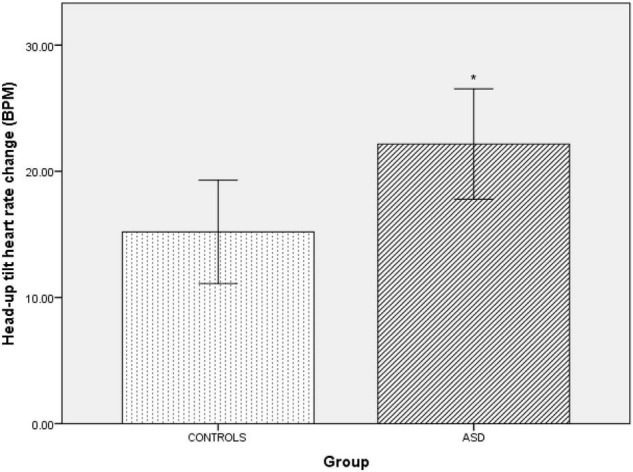
Changes in heart rate from baseline during head-up tilt in controls and ASD. BPM, beats per minute. *Refer to a statistically significant finding.

In the ASD group during head-up tilt, there was a significant (*t*(41) = –2.32, *p* = 0.025) increase in heart rate (Δ22 bpm ± 10.60) compared to the control group (Δ15 bpm ±8.24, Figure 6).

### Respiratory Sinus Arrythmia

Cardiovagal function was assessed by subtracting the minimum heart rate response from the maximum heart rate response to deep breathing averaged over a minimum of 5 breathing cycles. During paced deep breathing, respiratory sinus arrhythmia indicated significantly [*t*(30) = –3.60, *p* = 0.001] increased cardiac acceleration during inspiration in the ASD group (93 bpm + 13.59) compared to the healthy control group (72 bpm ± 12.27). Vagally-mediated cardiac deceleration during expiration in the ASD group was significantly [*t*(30) = –3.58, *p* = 0.001] attenuated (75 bpm ± 14.31) compared to the healthy control group (55 bpm ± 10.07).

### Valsalva Maneuver

**FIGURE 7:**
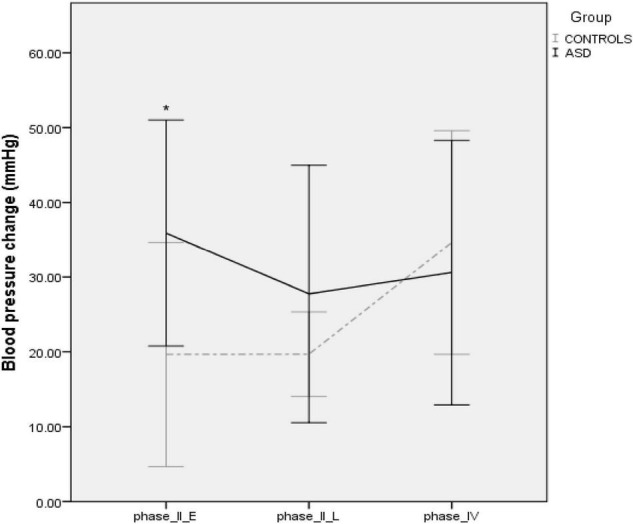
Blood pressure profiles during the Valsalva maneuver in controls and ASD. mmHg, millimeters of mercury. *Refer to a statistically significant finding.

Four ASD subjects had a normal variant flat top BP response (lack of arterial pressure decrease during phase II early) to Valsalva maneuvers. One patient was unable to follow the guidance required to perform the Valsalva maneuver and was therefore excluded from analysis. During phase II early of the Valsalva maneuver, the typical fall in blood pressure due to reduced venous return and stroke volume was significantly [*t*(19) = –2.23, *p* = 0.037] attenuated in the ASD group (Δ36 mmHg ± 15.42) compared to the control group (Δ19 mmHg ± 1, Figure 7).

### Autonomic Diagnoses

20/28 (71%) ASD subjects had a recognizable autonomic disorder (Table 1). 9 ASD had PoTS, 4 had PoTS and VVS, 3 experienced presyncope (i.e., testing was aborted as the blood pressure and heart rate profiles indicated they were about to faint), 1 had VVS, 1 had (generalized) essential hyperhidrosis, 1 had orthostatic hypotension and 1 had comorbid PoTS, VVS and essential (predominantly craniofacial) hyperhidrosis. Of the 16/20 (80%) ASD patients with an autonomic disorder also had hE-DS.

### Psychiatric Co-morbidities

These are listed, with diagnosis and the numbers affected. One had a pre-existing diagnosis of severe cognitive impairment, 1 a pre-existing diagnosis of learning difficulties, 1 a pre-existing diagnosis of anxiety disorder, 1 a pre-existing diagnosis of obsessive-compulsive disorder, self-harm, 1 a pre-existing diagnosis of Tourette’s syndrome, 1 a pre-existing diagnosis of schizophrenia, 1 a pre-existing diagnosis of depression and 2 with a pre-existing diagnosis of anxiety disorder.

## Discussion

This is the first study of detailed cardiovascular and thermoregulatory autonomic testing in ASD in two national referral centers for autonomic disorders. The association with hE-DS being over-represented in ASD and orthostatic intolerance also was determined. In ASD, 20/25 (80%) had an autonomic condition; 9 had PoTS, 4 had PoTS and VVS, 3 experienced presyncope (i.e., testing aborted due to the beat-to-beat blood pressure and heart rate indicating they were about to faint) during standing or HUT, 1 had VVS, 1 had generalized essential hyperhidrosis, 1 had orthostatic hypotension and 1 had PoTS, VVS and essential (craniofacial) hyperhidrosis. 80% of ASD with dysautonomia had hE-DS.

Resting heart rate was significantly higher in ASD, with a greater increase during HUT, favoring enhanced sympathoexcitation. During phase II early of the Valsalva maneuver, the fall in blood pressure was significantly reduced in ASD compared to controls. This differs from the typical Valsalva profile in PoTS (Aman, 2004) with a trend toward flat top Valsalva profiles and complements the probably increased sympathetic tone during baseline. PoTS subjects also may have hypovolemia, which could reduce cardiac output. Whether this may occur in is unclear.

Pressor responses were blunted in ASD, unlike increased cardiac sympathoexcitation during HUT in this group, and this is of relevance to β-adrenergic antagonists that are reported to reduce some of the negative common behaviors, such as aggression and impulsiveness in ASD (Boddaert et al., 2004). The attenuated diastolic blood pressure (representing peripheral resistance) responses to isometric exercise, cold pressor and mental arithmetic in ASD also may relate to the prevalence of hE-DS, where there may be greater distensibility of blood vessels (with the collagen deficit), thus diminishing vasoconstriction during pressor tests, and predisposing to orthostatic intolerance.

Neuroimaging studies have offered insights into potential central reasons for perturbed autonomic function in ASD. Both ASD and hE-DS are associated with reductions in right superior temporal volume, an area vital for the sensory processing of social emotional information (Boddaert et al., 2004), and in amygdala development (Eccles et al., 2012; Bernhardt et al., 2016). The amygdala is a key structure in the central autonomic network and its activity, along with the dorsal anterior cingulate cortex, predicts changes in heart rate (Jänig and Häbler, 2003) and cardiac contractility during stress (Dalton et al., 2005). Moreover, in ASD, altered functional connectivity and activation (Hadjikhani et al., 2009; Ebisch et al., 2011; Watanabe et al., 2012; Duerden et al., 2013) during emotional processing of the insular, another part of the central autonomic network (Bennaroch, 1993), has been reported. The anterior insular cortex (AIC) contains a significant number of “von Economo neurons,” which are large bipolar, spindle-shaped projection neurons (Seeley et al., 2012). These neurons are prevalent in humans and are mainly situated in layer Vb of the anterior cingulate cortex and the fronto-insular cortex (i.e., the junction of AIC and posterior orbito-frontal cortex) and are specifically associated with efferent autonomic mediation to the brainstem and spinal cord (Evrard, 2019) and afferent visceral feedback (known as, “interoception”) (Allman et al., 2011). In comparison to controls, ASD have von Economo abnormalities, morphological differences (Simms et al., 2009) and a significantly greater ratio of von Economo neurons to pyramidal neurons (Santos et al., 2011), of probable relevance to autonomic disturbances reported here, and interoceptive sensitivity reported previously in ASD (Quattrocki and Friston, 2014).

The psychiatric comorbidities in some with ASD are likely to relate to ASD itself rather than the anxiety symptoms reported in PoTS, VVS and essential hyperhidrosis, which are typically sub-clinical and specific to somatic hypervigilance (Eccles et al., 2015; Owens et al., 2016, 2017). Two ASD subjects, one with PoTS and another with generalized hyperhidrosis were cognitively impaired and their autonomic symptoms were pronounced enough to be noted by their caregivers, hence their referral for autonomic investigation. This may be of relevance to some of their symptoms and needs consideration in their overall management. The co-occurrence of ASD and hE-DS has only fairly recently (since 2014) (Shetreat-Klein et al., 2014) been formally reported in a well-characterized dataset. Overlapping symptoms include, anxiety, pain, sleep disruption and gastrointestinal problems (Tinkle et al., 2017). And this study builds on our previous findings investigating the prevalence of neurodevelopmental disorders in those with autonomic dysfunction and hE-DS (Sandroni et al., 2000). The current findings draw together the previously separate investigations of ASD and hE-DS with those exploring ASD and autonomic function, and indicates these two currently separate bodies of work could be drawn together to elucidate the overlap and interplay between ASD and autonomic dysfunction, which should benefit diagnostic evaluation and treatment strategies.

This retrospective study was in a relatively small sample of ASD and studies in larger numbers are required to provide further in-depth analyses of ANS function/dysfunction in ASD. The current data also was collected from two national referral centers for autonomic conditions, with subjects referred because of possible autonomic dysfunction. Further studies in a larger cohort with ASD, regardless of autonomic symptoms is needed. There are clear differences between ASD and controls, with 16/20 (80%) of ASD having a confirmed autonomic condition, also with hE-DS suggesting that further examination of the overlapping relationship between ASD, autonomic dysfunction and hE-DS may be informative, particularly in relation to biomarker identification. Additional studies with larger numbers, a broader spectrum of autonomic assessments and detailed autonomic evaluation should provide further in-depth analysis of ANS function and dysfunction in ASD, and the relationship to their key issues. Studies in ASD that are apparently asymptomatic with autonomic dysfunction are likely to provide further information.

## Conclusion

In this study elevated heart rate while supine and upright was present in ASD in comparison to controls, suggestive of increased sympathoexcitation. Sympathetic vasoconstriction, however, appeared impaired in ASD. Intermittent autonomic dysfunction (cardiovascular and thermoregulatory) was over-represented in ASD, and there was a strong association with hE-DS. Abnormalities in the central autonomic network may be contributory and further compromise autonomic function. Autonomic dysfunction may impair quality of life in ASD, particularly in those unable to express their experience of such symptoms. The interaction of autonomic symptoms with cognitive-affective and socio-emotional symptomatology warrants further investigation in larger groups with ASD, including those initially with no apparent autonomic features.

## Data Availability Statement

The raw data supporting the conclusions of this article will be made available by the authors, without undue reservation.

## Ethics Statement

The studies involving human participants were reviewed and approved by London—Harrow Research Ethics Committee. The patients/participants provided their written informed consent to participate in this study.

## Author Contributions

AO, CM, and VI: conception and design of study, analysis, interpretation of data, and drafting and revising manuscript. AO: acquisition of data. All authors contributed to the article and approved the submitted version.

## Conflict of Interest

The authors declare that the research was conducted in the absence of any commercial or financial relationships that could be construed as a potential conflict of interest.

## Publisher’s Note

All claims expressed in this article are solely those of the authors and do not necessarily represent those of their affiliated organizations, or those of the publisher, the editors and the reviewers. Any product that may be evaluated in this article, or claim that may be made by its manufacturer, is not guaranteed or endorsed by the publisher.
